# A HIF1α-GPD1 feedforward loop inhibits the progression of renal clear cell carcinoma via mitochondrial function and lipid metabolism

**DOI:** 10.1186/s13046-021-01996-6

**Published:** 2021-06-07

**Authors:** Ren Liu, Yuanfa Feng, Yulin Deng, Zhihao Zou, Jianheng Ye, Zhiduan Cai, Xuejin Zhu, Yingke Liang, Jianming Lu, Hui Zhang, Yong Luo, Zhaodong Han, Yangjia Zhuo, Qingling Xie, Chi Tin Hon, Yuxiang Liang, Chin-Lee Wu, Weide Zhong

**Affiliations:** 1 Guangdong Provincial Institute of Nephrology, Nanfang Hospital, Southern Medical University, Guangzhou, 510515 China; 2 Department of Urology, Guangdong Key Laboratory of Clinical Molecular Medicine and Diagnostics, Guangzhou First People’s Hospital, School of Medicine, South China University of Technology, Guangzhou, 510180 China; 3 Urology Key Laboratory of Guangdong Province, The First Affiliated Hospital of Guangzhou Medical University, Guangzhou Medical University, Guangzhou, 510230 China; 4 Department of Urology, the Fifth Affiliated Hospital of Guangzhou Medical University, Guangzhou, 510700 China; 5 Department of Urology, Affiliated Foshan Hospital of Southern Medical University, Southern Medical University, Foshan, 528000 China; 6 Macau Institute of Systems Engineering, Macau University of Science and Technology, Avenida Wai Long, Taipa, Macau, 999078 China; 7 Departments of Urology, Massachusetts General Hospital and Harvard Medical School, Boston, MA 02114 USA; 8 Department of Pathology, Massachusetts General Hospital and Harvard Medical School, Boston, MA 02114 USA

**Keywords:** Hypoxia, ccRCC, Metabolism, HIF1α, GPD1

## Abstract

**Background:**

Hypoxia signaling, especially the hypoxia inducible factor (HIF) pathway, is a major player in clear cell renal cell carcinoma (ccRCC), which is characterized by disorders in lipid and glycogen metabolism. However, the interaction between hypoxia and lipid metabolism in ccRCC progression is still poorly understood.

**Methods:**

We used bioinformatic analysis and discovered that glycerol-3-phosphate dehydrogenase 1 (GPD1) may play a key role in hypoxia and lipid metabolism pathways in ccRCC. Tissue microarray, IHC staining, and survival analysis were performed to evaluate clinical function. In vitro and in vivo assays showed the biological effects of GPD1 in ccRCC progression.

**Results:**

We found that the expression of GPD1 was downregulated in ccRCC tissues, and overexpression of GPD1 inhibited the progression of ccRCC both in vivo and in vitro. Furthermore, we demonstrated that hypoxia inducible factor-1α (HIF1α) directly regulates GPD1 at the transcriptional level, which leads to the inhibition of mitochondrial function and lipid metabolism. Additionally, GPD1 was shown to inhibit prolyl hydroxylase 3 (PHD3), which blocks prolyl-hydroxylation of HIF1α and subsequent proteasomal degradation, and thus reinforces the inhibition of mitochondrial function and phosphorylation of AMPK via suppressing glycerol-3-phosphate dehydrogenase 2 (GPD2).

**Conclusions:**

This study not only demonstrated that HIF1α-GPD1 forms a positive feedforward loop inhibiting mitochondrial function and lipid metabolism in ccRCC, but also discovered a new mechanism for the molecular basis of HIF1α to inhibit tumor activity, thus providing novel insights into hypoxia-lipid-mediated ccRCC therapy.

**Supplementary Information:**

The online version contains supplementary material available at 10.1186/s13046-021-01996-6.

## Background

Renal cancer is a common urologic cancer, and the pathological classification is diverse. It consists mainly of renal clear cell carcinoma, most of which occurs in renal tubular epithelial cells [1]. Clear cell renal cell carcinoma (ccRCC) is histologically defined as malignant renal tubular epithelial cells with transparent and bright cytoplasm due to the massive accumulation of lipids and glycogen [2], suggesting that altered lipid and glucose metabolism plays a major role in its progression. From an epigenetic point of view, the main feature of ccRCC is the biallelic loss of the von Hipper Lindau (VHL) tumor suppressor. VHL encodes an E3 ubiquitin ligase that degrades hypoxia inducible factors (HIF) 1α and HIF2α [3], which leads to deregulation of the hypoxia pathway. In contrast to many tumor types, HIF1α and HIF2α have opposite roles in ccRCC biology, where HIF1α acts as a tumor suppressor and HIF2α acts as an oncogene [4]. This has promoted the development of HIF2α-specific inhibitors, which exhibit exceptional targeting effects in ccRCC xenograft models and show efficacy in a subset of patient-derived xenograft models and clinical responses in some phase I clinical trials [5]. While there are many studies focusing on the mechanism underlying the HIF2α-dependent promotion of ccRCC progression, studies regarding the mechanism of how HIF1α inhibits tumors are insufficient. Thus far, only one study by Florinda et al. has shown that HIF1α functions as an inhibitor of aspartate biogenesis by repressing glutamine oxidation and reductive carboxylation pathways via negatively regulating two key players, cytosolic glutamic-oxaloacetic transaminase-1 (GOT1) and mitochondrial GOT2 [6].

Proline hydroxylase (PHD) enzymes catalyze the oxygen-dependent hydroxylation of specific proline residues of HIFs, thereby causing binding to pVHL, ubiquitination, and subsequent proteasomal degradation. Under hypoxic conditions, the PHD enzymes are inhibited. As a result, HIF-α escapes degradation and forms a dimer with the HIF-1β subtype, which is expressed constitutively. This complex is then transported into the nucleus and binds to the hypoxia response element (HRE) within the promoter region upstream of the coding region, thereby affecting the transcription of a variety of genes, which also leads to lipid reprogramming and other metabolic disorders [7]. Lipidomic studies have demonstrated that fatty acids, triglycerides, and cholesterol exist at higher levels in ccRCC tissues as compared with normal kidney tissues [8]. Therefore, hypoxia-lipid-pathways play a significant role in the progression of ccRCC, but the underlying mechanism is not clear.

In this study, we analyzed and screened the RNA-seq data of 530 ccRCC patients in The Cancer Genome Atlas (TCGA) from two aspects, hypoxia and lipid metabolism, using bioinformatic analysis. We found that GPD1 was significantly involved in hypoxia and lipid metabolism pathways in the progression of ccRCC. Glycerol-3-phosphate dehydrogenase 1 (GPD1), catalyzes the reaction of acetone dihydrogen phosphate (DHAP) and nicotinamide adenine dinucleotide (NADH) into glycerol-3-phosphate (G3P) and nicotinamide adenine dinucleotide (NAD+), which provides substrates for the synthesis of glycerol, and links lipogenesis and carbohydrate metabolism [9]. GPD1 and GPD2 isoenzymes together form the glycerol triphosphate shuttle system. GPD1 functions in the cytoplasm, and GPD2 catalyzes the reversible conversion in the mitochondria and transfers electrons to the oxidative respiratory chain to promote aerobic phosphorylation [10, 11]. Studies have shown that knocking down the expression of GPD2 can induce AMPK phosphorylation [12] and inhibit mitochondrial functioning [13], which results in a reduction of energy supply and the suppression of tumors. Our previous study also found that the overexpression of GPD1 in prostate cancer and lung cancer cells inhibits the aerobic phosphorylation of mitochondria, which may be related to the deregulation of the hypoxia pathway [14]. Another study reported that GPD1 was minimally expressed in ccRCC cancer tissues via proteomics [15], but there is a lack of in-depth research on the specific functions and mechanisms.

## Materials and methods

### Patients and tissues

This study was approved by the Human Ethics Committee of the Public Health Department of the People’s Republic of China and the Massachusetts General Hospital. All patients signed an informed consent form. According to relevant laws and legal standards, all samples were processed anonymously.

The 17 pairs of matched frozen samples of renal cell carcinoma and benign renal tissue adjacent to the cancer, as well as the human ccRCC tissue microarrays (TMAs) used in this study, which contained 69 case samples, were all from the Massachusetts General Hospital. The inclusion criterion was that the patient did not receive chemotherapy or radiotherapy before surgery. Each case was diagnosed and graded by two pathologists separately and re-examined by HE staining. Relevant clinical information, including age, gender, Fuhrman grade, AJCC (American Joint Committee on Cancer) tumor T stage, tumor size, metastasis status, and overall survival time, was collected. The overall survival was defined as the time from the date of surgery to the last follow-up or death.

### Cell culture

All human cell lines were purchased from ATCC (American Type Culture Collection, Manassas, Virginia, USA), including HK2, RCC4, 769P, ACHN, Caki1, and Caki2. All cell lines were authenticated by polymorphic short tandem repeat profiling and were cultured at 37 °C in a 5% CO_2_ incubator with appropriate cell culture medium according to ATCC guidelines (**supplementary materials**). Hypoxia cell culture was achieved in a hypoxia chamber flushed with a pre-analyzed gas mixture of 1% O_2_, 5% CO_2_, and 95% N_2_ at 37 °C for 24 h.

### Construction and transfection of cell lines

To construct the lentiviral plasmids, an encoding vesicular stomatitis virus was used to envelope the protein plasmid pMD.2G (5 μg) and packaging plasmid psPAX2 (10 μg) by using the calcium phosphate method. Then, HEK293 cells were transfected with the lentiviral plasmids (12 μg), and the supernatant virus solution was collected after 48 h. Caki1 and 769P cells were seeded in 12-well plates and transfected with the supernatant virus solution for 12 h and incubated for another 24 h with fresh medium. Via flow cytometric analysis, we observed the expression of GFP to confirm the transfection. We collected the expanded cultured tumor cells, performed flow sorting, and enriched GFP+ tumor cells through multiple sortings, such that GFP+ cells were more than 99%, and the expression was stable. Through western blotting, we identified the Caki1 and 769P cell lines that stably overexpressed GPD1.

Human GPD1-specific siRNA, human GPD2-specific siRNA, human HIF1α-specific siRNA, human HIF2α-specific siRNA, and negative control siRNA were purchased from GenePharma (Suzhou, China). The siRNA sequences are shown in the **supplementary materials**. Western blotting analyses were conducted 48 h after transfection to test the transfection efficacy at the protein level.

### qRT-PCR

Quantitative analysis of mRNA expression was performed using qRT-PCR according to our published protocols [14, 16]. Sequences of all primers used for PCR are provided in the **supplementary materials**.

### Western blot analysis

Quantitative analysis of protein expression in cell lines and clinical tissues was performed using western blotting according to the protocol of our previous studies [14, 16]. A list of antibodies is provided in the **supplementary materials**.

### Animals and xenografts

All animal experiments were performed in accordance with the guidelines of the Institute for Laboratory Animal Research and the National Cancer Center Research Institute. Four-to-five-week-old BALB/c nude male mice were purchased from the Experimental Animal Center of Sun Yat-sen University (Guangzhou, China). For each xenograft assay, 5 × 10^6^ cells were subcutaneously injected into the flanks of nude mice. Tumor sizes and body weights were measured every three days. Tumor volumes were determined by the following formula: (L × W^2^)/2.

### Immunohistochemistry

GPD1 protein expressions in ccRCC tissues and normal kidney tissues were detected by immunohistochemistry (IHC) according to our previous published protocols [14, 16]. The GPD1 antibody was purchased from Santa Cruz (sc-376,219, Dallas, Texas, USA).

### Cell proliferation and migration analyses

Cell proliferation was detected by a CCK-8 (Cell Counting Kit-8, MA0218, Meilunbio, Dalian, China) assay. Cell migration was detected by a wound healing assay as previously described [14, 16].

### Cell cycle and apoptosis analyses

Cell cycle analysis was performed using the Cell Cycle Staining kit (70-CCS012, Multi Sciences, Hangzhou, China) according to the manufacturer’s protocol. For the cell apoptosis analysis, the Annexin V-FITC/PI Apoptosis Detection Kit (70-AP101–100, Multi Sciences) was used according to the manufacturer’s instructions. The stained cells were washed with PBS and analyzed via flow cytometry on a CytoFLEX system (CytoFLEX S, Beckman Coulter, Brea, California, USA).

### Seahorse assay

Cells were seeded (8 × 10^3^ cells/well) in an XFe24 cell culture microplate (100,777, Agilent, Santa Clara, California, USA). We used the XFe24 Cell Mito Stress Test (100,850, Agilent) to determine mitochondrial function by directly measuring the oxygen consumption rate (OCR) of each cell line as described in our previous studies [14, 16]. The XF ATP Rate Assay (103,592, Agilent) was used to simultaneously measure the rate of ATP production and was run in an XFe24 Extracellular Flux Analyzer (Agilent). The data were analyzed using the Wave software program 2.6.0 (Agilent).

### Immunofluorescence

Cells were seeded in 6-well plates, treated with 4% formaldehyde for 15 min, and then homogenized in 0.5% Triton X-100 for 15 min. Next, the primary antibodies, GPD1 (sc-376,219, Santa Cruz) and HIF1α (20960–1-AP, Proteintech, Wuhan, China), were diluted in 5% BSA respectively and incubated with the cells overnight at 4 °C. After that, the cells were washed three times with PBS and then incubated with a second antibody (Alexa Fluor, Boster, Guangzhou, China) at 25 °C for 1 h. Finally, a DAPI antibody was incubated with the cells for 5 min, followed by washing three times and the addition of the fluorescence quenching agent. The cells were imaged using a confocal laser scanning microscope (LSM880, Leica, Germany).

### CUT&RUN

CUT&RUN experiments were conducted using 300,000 RCC4 cells with the CUT&RUN assay kit (#86652, Cell Signaling Technology, Beverly, MA, USA). In brief, cells were washed and bound to concanavalin A-coated magnetic beads, and then performed permeabilization was performed with Wash Buffer containing 0.05% digitonin. After that, cells were incubated with 5 μg of HIF1α antibody (ab1, Abcam, Shanghai, China) for 8 h at 4 °C. Then, cell-bead slurry was washed twice with Dig Wash Buffer containing 0.05% digitonin and incubated with Protein A-MNase for 1 h at 4 °C. After washing with Wash Buffer containing 0.05% digitonin, 2 mM CaCl_2_ was added into the cell-bead slurry to initiate Protein A-MNase digestion, which was then incubated for 30 min on ice. After the antibody-specific incubation period, the reaction was stopped with the addition of one volume of 2x Stop Buffer containing 20 mM EDTA, 0.05% digitonin, 5 mg/ml RNase A, and 2 pg/ml heterologous spike-in DNA. CUT&RUN fragments were released by incubation for 30 min at 37 °C followed by centrifugation for 5 min at 16,000×*g*. After centrifugation, the supernatant was recovered, and the DNA extracted using DNA Purification Buffers and Spin Columns (#14209, Cell Signaling Technology). The resulting DNA products were quantified by qRT-PCR as described above. The primers used for qRT-PCR amplification are described in the **supplementary materials****.**

### Luciferase reporter assay

The HIF1α plasmid, firefly luciferase plasmid GPD1-HBS promoter, firefly luciferase plasmid GPD2-HBS1 plus HBS2 promoter, and firefly luciferase plasmid GPD2-HBS2 promoter were all purchased from GenePharma (Suzhou, China) along with the renilla luciferase plasmid. HEK293 cells were grown to a density of 5000 cells per well in a 96-well plate and transfected with the renilla luciferase plasmid, HIF1α plasmid, and firefly luciferase plasmid with the indicated promoter region. After 48 h, luciferase activity was measured by using the Firefly & Renilla Luciferase Reporter Assay Kit (MA0518, Meilunbio, Dalian, China) and a Varioskan Full Wavelength Fluorescence Microplate Reader (Thermo Fisher Scientific, Shanghai, China). The relative luciferase activity was calculated from the firefly luciferase normalized to renilla luciferase. The luciferase reporter assays were conducted in triplicate, and repeated independently for three times. The promoter sequences are listed in the **supplementary materials****.**

### Coimmunoprecipitation (Co-IP)

HEK293 cells were grown to 70% confluence in 6-well plates and then transfected with GPD1 or negative control plasmids for 24 h. Cell lysates were prepared for Co-IP analysis (#88805, Thermo Fisher Scientific). Briefly, beads were prewashed two times with 1× modified coupling buffer and then incubated with 5 μg of HIF1α antibody (ab8366, Abcam) for 15 min. After that, the beads were washed three times with 1× modified coupling buffer, and the antibody was crosslinked in 1 ml of disuccinimidyl suberate solution for 30 min. Then, the beads were washed three times with elution buffer, followed by two IP lysis/wash buffer washes. After that, the cell lysates were incubated with the beads for 8 h at 4 °C. The next day, the beads were washed twice with 1 ml of IP lysis/wash buffer, followed by a final wash with ultrapure water. The bound antigen was finally eluted and prepared for western blotting analysis.

### Oil red O staining

Cells were plated in six-well plates in triplicate, followed by rinsing with PBS twice. Then, the cells were fixed with 10% formaldehyde for 30 min. After that, 1 ml of 60% isopropanol per well was used to wash the cells, and 1 ml of 0.5% Oil Red O solution (MA0120, Meilunbio, Dalian, China) was incubated with the cells for 10 min, after which the cells were washed five times with water. The nuclei were stained with hematoxylin staining solution (MB9897, Meilunbio) and differentiated with 1% hydrochloric acid solution for 1 min. Filter paper was used to absorb the surrounding moisture, and then the slides were mounted with glycerin gelatin and observed under a microscope.

### Free fatty acid quantitation assay

A Free Fatty Acid Quantitation Kit was used (MAK044, Merck, Darmstadt, Germany) to quantify the free fatty acid levels. According to the protocol, cells (1 × 10^6^) were homogenized in 200 μl of 1% (w/v) Triton X-100 in chloroform. After centrifugation at 13,000×*g* for 10 min, samples in the lower organic phase were collected, air dried at 50 °C to remove chloroform, and then placed under a vacuum for 30 min to remove any organic solvent. The dried samples were then dissolved in 200 μl of fatty acid assay buffer by sonication and vortexed until the samples were homogeneous. Then, according to the manufacturer’s protocol, a reaction mix containing 2 μl ACS reagent, 44 μl fatty acid assay buffer, 2 μl fatty acid probe, 2 μl enzyme mix, and 2 μl enhancer was set up and incubated with samples for 30 min at 37 °C. Free fatty acid levels were then determined by colorimetric absorbance at 570 nm.

### Bioinformatic analysis

Data from 539 ccRCC patients and 72 paired kidney tissues were obtained from the TCGA database (The Cancer Genome Atlas, https://portal.gdc.cancer.gov). To detect the hypoxia- and lipid-related subtypes in TCGA-KIRC patients, dimensionality reduction and clustering was performed using the ‘Rtsne’ package and ‘kmeans’ function based on the hallmark gene sets of hypoxia- or lipid-related genes, respectively. The gene sets of ‘HALLMARK_HYPOXIA’ and ‘HALLMARK_FATTY_ACID_METABOLISM’ were obtained from the Molecular Signatures Database (MSigDB version 6.0). Kaplan-Meier analysis and the log-rank test were used to compare the overall survival outcomes in different clusters using the R package “survival”. Then, the hypoxia- and lipid-related differentially expressed genes (DEGs) were obtained by the expression comparison between different prognostic clusters. The intersection of hypoxia-related DEGs and lipid-related DEGs was defined as ‘hypoxia/lipid DEGs’. To further narrow down the significant features among hypoxia/lipid DEGs, the Least Absolute Shrinkage and Selection Operator (LASSO) method was further performed based on the R package ‘glmnet’ [17]. With the optimal lambda value, the candidate hypoxia/lipid DEGs were selected for further analysis. In addition, a hypoxia/lipid-related signature was constructed based on the selected candidate hypoxia/lipid DEGs. Risk stratification analysis, i.e. Kaplan-Meier analysis, Wilcoxon signed-rank test, and the time-dependent receiver operating characteristic (ROC) curve, were utilized to evaluate the predictive value of the hypoxia/lipid-related signature. All of the above bioinformatic analyses were performed in R (version 4.0.2).

The RNA-seq dataset (GSE146982, https: //www.ncbi.nlm.nih.gov/geo/) was analyzed using the GSEA (Gene Set Enrichment Analysis) software program ((http://www.broadinstitute.org/gsea).

### Statistical analysis

SPSS software (SPSS 23.0, SPSS Inc.) and GraphPad Prism 7 (https://www.graphpad.com/) were used for statistical analysis. Comparisons were performed by using a paired Student’s *t* test or variance analysis (ANOVA). Kaplan-Meier survival curves were used to assess survival time, and log-rank tests were used for comparison. Cox regression was performed for univariate and multivariate analyses. A *p* < 0.05 indicated that the difference was statistically significant.

## Results

### GPD1 serves an important role in hypoxia and lipid metabolism pathways in ccRCC

**Fig. 1:**
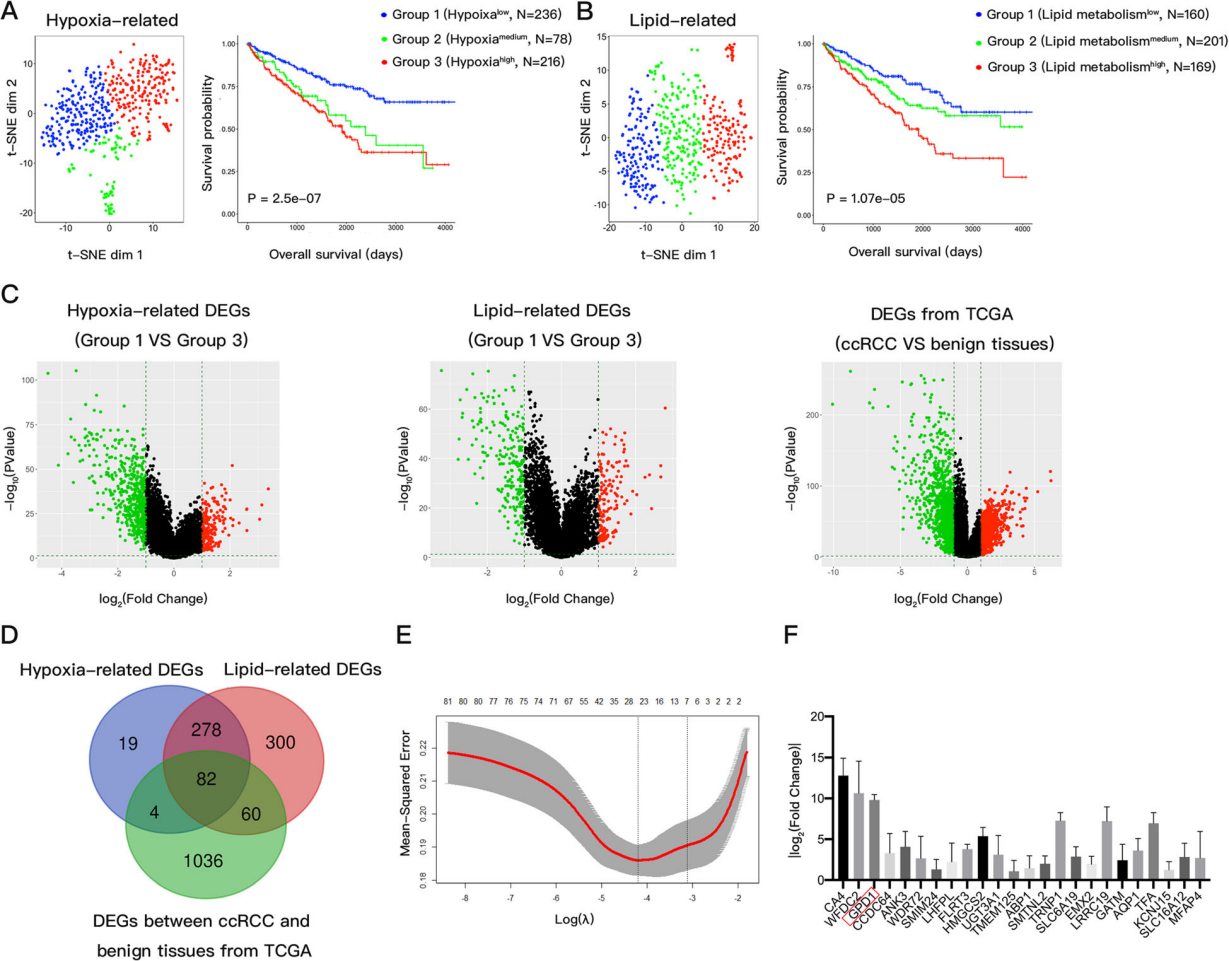
GPD1 serves an important role in hypoxia and lipid metabolism pathways in ccRCC. (**A**) Dot plots for groups (1–3) identified by a t-SNE algorithm based on hypoxic hallmark genes and Kaplan-Meier plots for overall survival. (**B**) Dot plots for groups (1–3) identified by a t-SNE algorithm based on lipid metabolism hallmark genes and Kaplan-Meier plots for overall survival. (**C**) Heatmaps showing expression profiles for hypoxia-related DEGs, lipid-related DEGs, and DEGs between ccRCC and benign kidney tissues in the TCGA database. (**D**) A Venn diagram showing overlapping hypoxia-related DEGs, lipid-related DEGs, and DEGs between ccRCC and benign kidney tissues in the TCGA database. (**E**) LASSO Cox regression model that selected 24 prognostic hypoxia-lipid-related DEGs. (**F**) Alterations in mRNA levels of 24 prognostic hypoxia-lipid-related DEGs under hypoxic conditions. Abbreviations: GPD1, glycerol-3-phosphate dehydrogenase 1; ccRCC, clear cell renal cell carcinoma; t-SNE, t-distributed stochastic neighbor embedding; DEGs, differently expressed genes; TCGA, The Cancer Genome Atlas; LASSO, least absolute shrinkage and selection operator

The discovery cohort contained 530 ccRCC patients from the TCGA database, including the corresponding RNA-seq results and clinical information. Based on the expression matrix of 200 hypoxia hallmark genes as defined by the Broad Institute Molecular Signatures database, a non-linear dimensionality reduction algorithm t-SNE was used to divide all patients into three groups (Groups 1, 2, and 3). A comparison of survival curves showed obvious significant differences among the three groups (log-rank test, *p* < 0.001) (Fig. 1A). The overall survival rates of the patients in Group 1 were significantly higher than those of the patients in Group 3, which indicated that the hypoxia status was lowest in Group 1 and highest in Group 3. We further divided all patients into three lipid-related groups using the same method based on the expression matrix of 200 lipid metabolism hallmark gene sets. Similarly, Group 1, with the best overall survival, was the low-lipid metabolite group, while Group 3, with the worst overall survival, was the high-lipid metabolite group (Fig. 1B). Through differential gene analysis (|log2FC| > 1, FDR-adjusted *p* < 0.01), 383 genes were found to be differentially expressed in the hypoxia^high^ (Group 3) and hypoxia^low^ (Group 1) groups, and there were 720 DEGs between the lipid metabolism^high^ (Group 3) and lipid metabolism^low^ (Group 1) groups (Fig. 1C). Next, we obtained 82 genes by overlapping the hypoxia-related DEGs, the lipid-related DEGs, and the DEGs between the ccRCC and benign tissues acquired from TCGA (|log2FC| > 1, FDR-adjusted p < 0.01) (Fig. 1D).

A LASSO Cox regression model was used to identify the most significant prognostic gene signature from these 82 hypoxia-lipid-related DEGs. The optimal gene signature consisted of 24 prognostic DEGs, and the corresponding coefficients were calculated (Fig. 1E and S1A). The ROC curve analysis suggested that the prognostic accuracy for these 24 genes was 0.8 (Fig. S1B). The distribution of risk score showed that patients who died had higher risk scores than patients who lived (*p* < 0.05). Using this 24-gene formula, the risk score of each patient in the TCGA cohort was calculated. The patients were then dichotomized equally into a high-risk group and a low-risk group according to the median risk score (Fig. S1C). Kaplan-Meier analysis showed that patients in the high-risk group had worse outcomes than those in the low-risk group (HR = 2.67, 95% CI 1.96 to 3.62, *p* = 1.20e-05) (Fig. S1D).

We found that 9 out of these 24 genes were previously studied in ccRCC [18–26]. We then cultured the ccRCC cell line 769P under normoxic and hypoxic conditions and assessed the expressions of the 24 genes, which were *CA4, WFDC2, GPD1, CCDC64, ANK3, WDR72, SMIM24, LHFPL, FLRT3, HMGCS2, UGT3A1, TMEM125, ABP1, SMTNL2, TRNP1, SLC6A19, EMX2, LRRC19, GATM, AQP1, TFA, KCNJ15, SLC16A12 and MFAP4*. We found that the mRNA expression of *GPD1* was the one of the most significantly altered under hypoxic conditions (Fig. 1F).

### The expression of GPD1 was downregulated in ccRCC tissues and inhibited the progression of ccRCC in vitro and in vivo

**Fig. 2:**
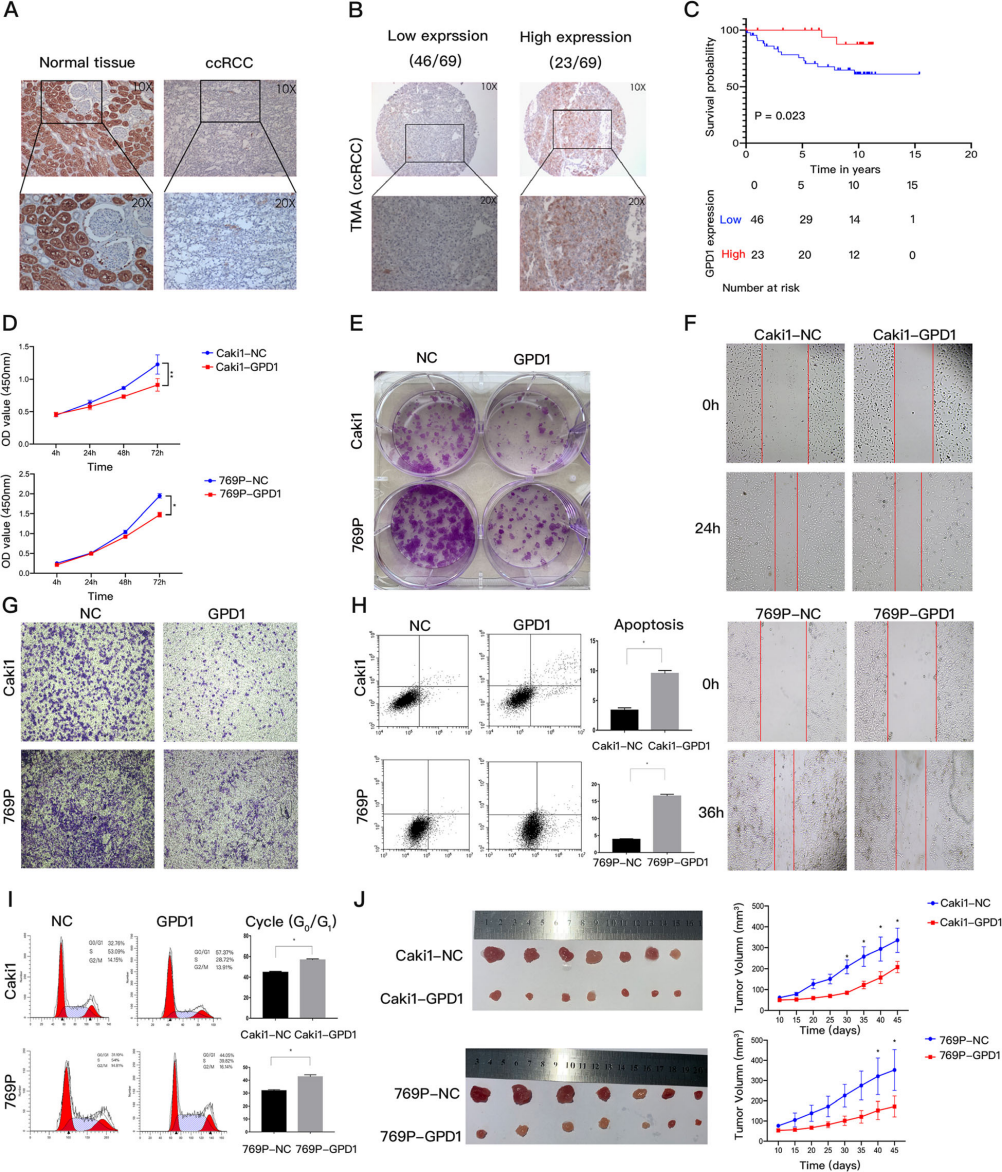
The expression of GPD1 was downregulated in ccRCC tissues and inhibited the progression of ccRCC in vitro and in vivo. (**A**) Immunostainings of GPD1 protein in ccRCC tissues and paired normal kidney tissues. (**B**) Immunostainings of GPD1 protein in 69 ccRCC samples of TMA. (**C**) Kaplan-Meier curves for overall survival between the GPD1 high-expression group and the GPD1 low-expression group based on GPD1 IHC scores (8–12, GPD1 high-expression, n = 23 vs. 0–6, GPD1 low-expression, n = 46). (**D-E**) Cell proliferation as determined by CCK-8 and colony formation assays. (**F-G**) Cell migration and invasion as determined by wound healing and Transwell assays. Red lines denote the margins of the wound. (**H**) Cell apoptosis analysis of Caki1 and 769P cells overexpressing GPD1 or a negative control. (**I**) Cell cycle analysis of Caki1 and 769P cells overexpressing GPD1 or a negative control. The results are presented as the mean + SD of three independent experiments. (**J**) Images of tumors from the subcutaneous xenograft models implanted using Caki1 and 769P cells that stably overexpressed GPD1 or a negative control. The growth curves of tumors are shown (*p < 0.05, **p < 0.01). Abbreviations: GPD1, glycerol-3-phosphate dehydrogenase 1; ccRCC, clear cell renal cell carcinoma; TMA, tissue microarray; IHC, immunohistochemistry; TCGA, the cancer genome atlas; CCK-8, cell counting kit

To investigate the expression and clinical significance of GPD1, we found that *GPD1* mRNA was expressed less in ccRCC tissues as compared to normal kidney tissues based on the TCGA-KIRC database (*p* < 0.01) (Fig. S2A). The protein expression of GPD1 in ccRCC and normal tissues was examined by IHC and western blotting. We found that GPD1 protein was mainly expressed in the cytoplasm of renal tubular epithelial cells, and the expression level in benign tissues was much higher than that in tumor tissues (Fig. 2A and S2B).

The associations between GPD1 expression and the clinical pathological characteristics of ccRCC patients based on our TMA data are shown in Table 1 (Fig. 2B). The expression of GPD1 was significantly associated with vital status (alive) (*p* = 0.039); however, there was no significant association between GPD1 expression and tumor stage, Fuhrman grade, or tumor size. A survival comparison showed that high GPD1 protein expression correlated with significantly better overall survival (log-rank test, *p* = 0.023) (Fig. 2C). Further univariate and multivariate Cox regression analyses revealed that GPD1 was an independent predictor of overall survival, which indicated that GPD1 is a favorable prognostic indicator (Table 2).

**Table 1:** Association between expression of GPD1 and clinical characteristics in ccRCC patients

Characteristic	GPD1		*P*-value
	Low (*n* = 46)	High (*n* = 23)	
Age (years)			0.097
< =65	32	20	
> 65	14	3	
Sex			0.832
Female	9	5	
Male	37	18	
Fuhrman grade			0.302
1/2	29	11	
3/4	17	12	
Tumor stage			0.748
I/II	38	18	
III/IV	8	5	
Metastasis			0.840
M0	35	18	
M1	11	5	
Size (longest diameter, cm)			0.158
< =7	33	20	
> 7	13	3	
Vital status (at followed-up)			0.039*
Alive	31	21	
Dead	15	2	

* P-value < 0.05

**Table 2:** Univariate and multivariate analysis of different prognostic parameters in ccRCC patients

Variables	Univariate analysis			Multivariate analysis		
	HR	CI	P-value	HR	CI	P-value
Age	2.951	1.135–7.671	0.026*	3.487	1.155–10.525	0.027*
Sex	0.656	0.214–2.017	0.462	0.329	0.095–1.140	0.080
T stage	1.056	0.303–3.678	0.932	0.543	0.131–2.247	0.399
Grade	1.843	0.701–4.845	0.215	4.198	0.854–20.638	0.077
Metastasis	2.542	0.965–6.693	0.059	2.957	0.969–9.024	0.057
Size	2.261	0.834–6.130	0.109	0.719	0.149–3.456	0.680
GPD1	0.213	0.049–0.930	0.040*	0.124	0.018–0.868	0.036*

* P-value < 0.05

We examined the expression of GPD1 in renal tubular epithelial cells (HK2) and various renal ccRCC cell lines and found that GPD1 was generally expressed at a lower level in tumor cell lines (Fig. S2C). Therefore, we established the GPD1-overexpressing stable ccRCC cell lines, Caki1 and 769P, via lentivector transduction and examined the expression via western blotting and qRT-PCR analyses (Fig. S2D). A CCK-8 assay showed that overexpression of GPD1 inhibited cell proliferation in both Caki1 and 769P cells (Fig. 2D). The colony formation abilities of GPD1-overexpressing ccRCC cells were greatly attenuated compared with the negative control groups (Fig. 2E and S2E). The wound healing and Transwell assays indicated that overexpression of GPD1 protein suppressed the migration and invasion of Caki1 and 769P cells (Fig. 2F, G and S2F). The cell cycle assay indicated that overexpression of GPD1 protein increased the percentage of G0/G1 phase cells (Fig. 2H). Moreover, we also performed flow cytometry analysis to determine the effect of GPD1 on apoptosis. The results showed that GPD1 significantly promoted apoptosis in Caki1 and 769P cells (Fig. 2I). Based on these results, we were interested in the role of GPD1 in tumor growth in vivo. As such, we tested the effect of Caki1 and 769P cell lines that stably expressed GPD1 in a murine xenograft model on tumor growth as compared to vector control cell lines. As shown in Fig. 2J, tumor growth was significantly inhibited by the overexpression of GPD1, and the tumors in the GPD1-overexpressing groups also had smaller sizes and lower weights than those in the control groups (Fig. S2G).

### Overexpression of GPD1 inhibited mitochondrial function and induced AMPK phosphorylation by suppressing the expression of GPD2

**Fig. 3:**
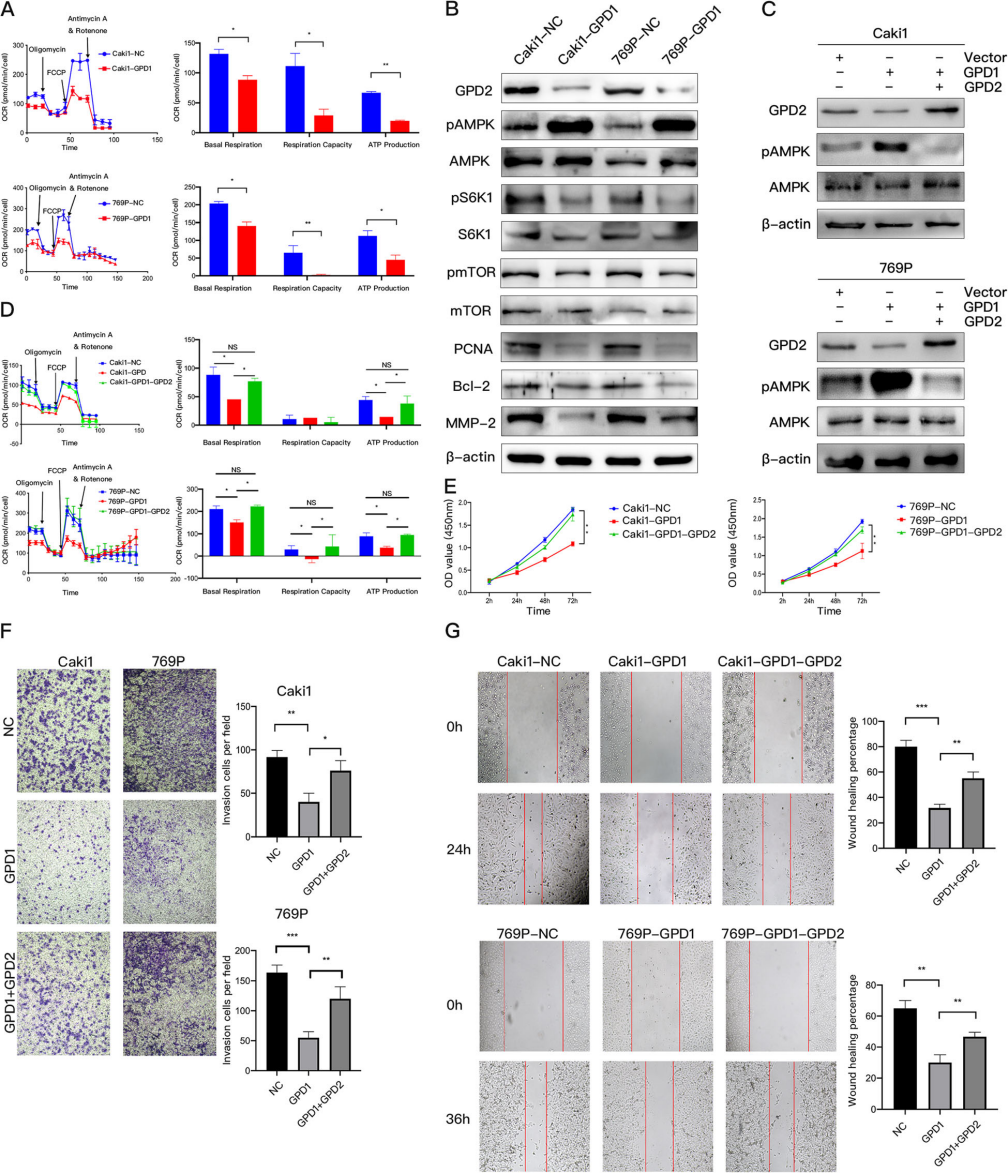
Overexpression of GPD1 inhibited mitochondrial function and induced AMPK phosphorylation by suppressing the expression of GPD2. (**A**) Oxygen consumption rate (OCR) of Caki1 and 769P cells with GPD1 overexpression. Baseline respiration, spare respiratory capacity, and ATP production in Caki1 and 769P cells with GPD1 overexpression. (**B**) Western blotting of indicated proteins in Caki1-NC, Caki1-GPD1, 769P -NC, and 769P-GPD1 cells. (**C**) Western blotting of GPD2, pAMPK, and AMPK in Caki1-GPD1 and 769P-GPD1 cells with GPD2 restoration and a negative control. (**D**) The OCR in Caki1-GPD1 and 769P-GPD1 cells with GPD2 restoration and a negative control. Baseline respiration, spare respiratory capacity, and ATP production in Caki1-GPD1 and 769P-GPD1 cells with GPD2 restoration and a negative control are shown. (**E**) Cell proliferation in Caki1-GPD1 and 769P-GPD1 cells with GPD2 restoration and a negative control as examined by CCK-8 assays. (**F–G**) Cell migration and invasion in Caki1-GPD1 and 769P-GPD1 cells with GPD2 restoration and a negative control as examined by wound healing and Transwell assays. Red lines denote the margins of the wound. Data are shown as the mean + SD (*n* = 3). Statistical analysis was performed using an unpaired Student’s *t* test with a two-tailed distribution (NS, *p* > 0.05, * *p* < 0.05, **p < 0.01, ***p < 0.001). Abbreviations: GPD1, glycerol-3-phosphate dehydrogenase 1; GPD2, glycerol-3-phosphate dehydrogenase 2; CCK-8, cell counting kit

We performed the seahorse assay for Caki1 and 769P cells with GPD1 overexpression to detect the OCR. The results showed that overexpression of GPD1 can significantly inhibited the levels of baseline respiration, ATP production, and spare respiratory capacity (Fig. 3A), which were consistent with the results after knocking down the expression of GPD2 (Fig. S3A and S3B). Western blotting analysis and qRT-PCR indicated that the protein and mRNA expression levels of GPD2 were significantly inhibited by the overexpression of GPD1. Additionally, AMPK phosphorylation was obviously induced, and the tumor mTOR pathway was suppressed (Fig. 3B and S3C). These results are consistent with the knock-out of GPD2 in previous studies [12]. Moreover, the expressions of PCNA (a proliferation marker), Bcl-2 (an apoptosis marker), and MMP-2 (an invasion marker) were both downregulated in ccRCC cells with GPD1 overexpression.

Therefore, we hypothesized that GPD1 may inhibit mitochondrial function, which affected the energy supply, by inhibiting the expression of GPD2 and then inducing AMPK phosphorylation, which could suppress the progression of ccRCC. To confirm this, we rescued the expression of GPD2 in Caki1 and 769P cells with the overexpression of GPD1. Rescue of GPD2 expression yielded a significantly weak phosphorylated AMPK signal (Fig. 3C) and restoration of mitochondrial function (Fig. 3D). Furthermore, restoration of GPD2 in Caki1-GPD1 and 769P-GPD1 cells significantly recovered their proliferation, migration, and invasion abilities (Fig. 3E, F, and G). However, the underlying regulatory mechanism is still unclear, and further research is needed.

### HIF1α directly targeted and positively regulated GPD1 and negatively regulated GPD2 at the transcriptional level

**Fig. 4:**
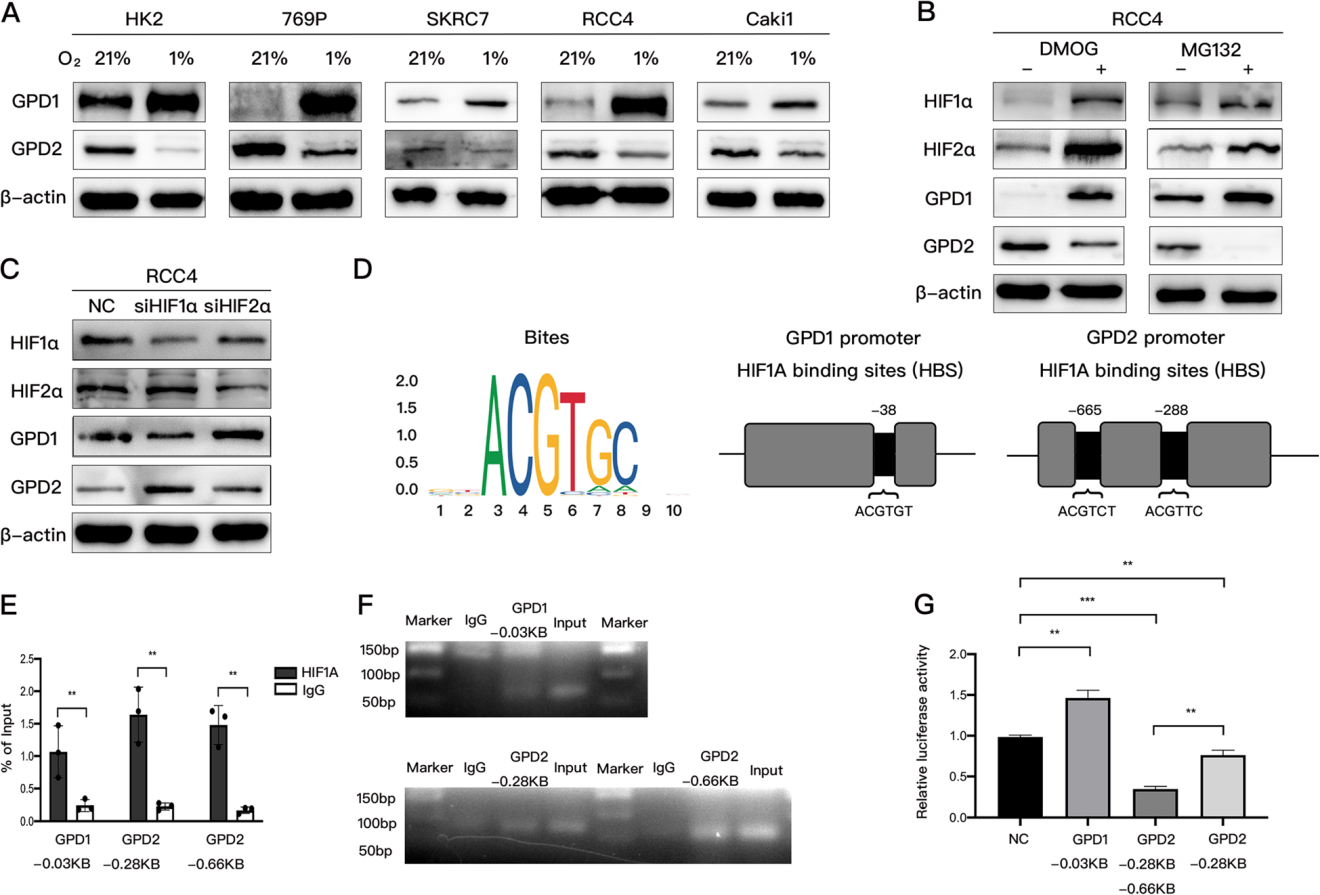
HIF1α directly targeted and positively regulated GPD1 and negatively regulated GPD2 at the transcriptional level. (**A**) HK2, 769P, SKRC7, RCC4, and Caki1 cells were exposed to 21% O_2_ or 1% O_2_ for 24 h, followed by examining GPD1 and GPD2 protein expression. (**B**) RCC4 cells were treated with DMOG (1 mmol/L) or MG132 (10 μmol/L) for 24 h, followed by western blotting. (**C**) RCC4 cells were transfected with HIF1α-specific siRNA (siHIF1α) or HIF2α-specific siRNA (siHIF2α) or negative control siRNA, followed by western blotting for indicated proteins. (**D**) Analysis of HIF1α binding sites (HBS) in human *GPD1* and *GPD2* promoters based on the JASPAR promoter database. (**E**) A CUT&RUN assay and qRT-PCR were performed with primers containing putative HBS sites in the *GPD1* or *GPD2* promoters. (**F**) GPD1 or GPD2 DNA were quantified using DNA agarose gel electrophoresis and qRT-PCR with different primers. (**G**) A luciferase reporter assay was performed in HEK293 cells transfected with the indicated promoter plasmids, including GPD1-HBS (− 38 bp), GPD2-HBS1 (− 665 bp), and GPD2-HBS2 (− 288 bp). Data are shown as the mean + SD (*n* = 3). Statistical analysis was performed using unpaired Student’s *t* test with a two-tailed distribution (*p < 0.05, **p < 0.01, ***p < 0.001). Abbreviations: HIF, hypoxia inducible factor; GPD1, glycerol-3-phosphate dehydrogenase 1; GPD2, glycerol-3-phosphate dehydrogenase 2; DMOG, dimethyloxalylglycine

Based on the above results, we hypothesized that GPD1 influenced the expression of GPD2 through the hypoxia pathway. To uncover the role of GPD1 and GPD2 under hypoxic conditions, HK2 cells and various renal ccRCC cell lines were exposed to hypoxia for 24 h (1% O_2_). We observed that the protein expression of GPD1 was significantly upregulated, while the protein expression of GPD2 was reduced (Fig. 4A). The mRNA expression of both *GPD1* and *GPD2* was similar to the protein expression (Fig. S4A). These observations indicated that GPD1 and GPD2 expression might be transcriptionally regulated after hypoxia exposure. To examine the possibility that GPD1 and GPD2 expressions were regulated by HIF transcription factors, we treated the ccRCC cell line RCC4 with a PHD inhibitor, dimethyloxalylglycine (DMOG), and a proteasome inhibitor, MG132, both of which stabilize HIFs. As shown in Fig. 4B, both DMOG and MG132 increased the expression of HIF1α and HIF2α, which might lead to the overexpression of GPD1 and inhibition of GPD2. Next, GPD1 expression was assessed in the ccRCC cell line RCC4 after siRNA-mediated knockdown of HIF1α and HIF2α. si-HIF1α significantly reduced the expression of GPD1 and increased the expression of GPD2, while si-HIF2α did not play any role (Fig. 4C).

To further validate whether HIF1α directly binds to the *GPD1* and *GPD2* promoter, the JASPAR database was analyzed for HIF-binding sites (HBS) within the *GPD1 and GPD2* gene promoter sequences. One candidate site at − 38 bp of *GPD1* and two candidate sites at − 288 bp and − 665 bp of *GPD2* were predicted (Fig. 4D). To test whether HIF1α binds at these sites to target *GPD1 or GPD2*, we performed a CUT&RUN assay in RCC4 cells. The results demonstrated the direct association of HIF1α with the *GPD1* and *GPD2* promoters (Fig. 4E and F). Furthermore, to verify whether the HBS within the *GPD1* and *GPD2* promoters functioned as hypoxia response elements (HRE), the fragment that encompassed the binding site of *GPD1* at − 38 bp upstream of the firefly luciferase-coding region was inserted into the pGL4 plasmid. The *GPD2* promoter region [700 bp before the transcription start site (TSS)] containing GPD2-HBS1 (− 665 bp) and GPD2-HBS2 (− 288 bp), as well as the *GPD2* promoter sequence (300 bp before TSS) containing only GPD2-HBS2 (− 288 bp), were both cloned upstream of the coding sequences for the firefly luciferase gene. Then, the luciferase reporter assay was performed and the results showed that GPD1-HBS significantly increased luciferase activity as compared to the negative control group. Additionally, GPD2-HBS1 plus HBS2 induced a significant reduction in luciferase activity as compared to the GPD2-HBS2 group or negative control group in HEK293 cells (Fig. 4G).

### GPD1 and HIF1α form a positive feedback loop to inhibit the expression of GPD2 and mitochondrial function

**Fig. 5:**
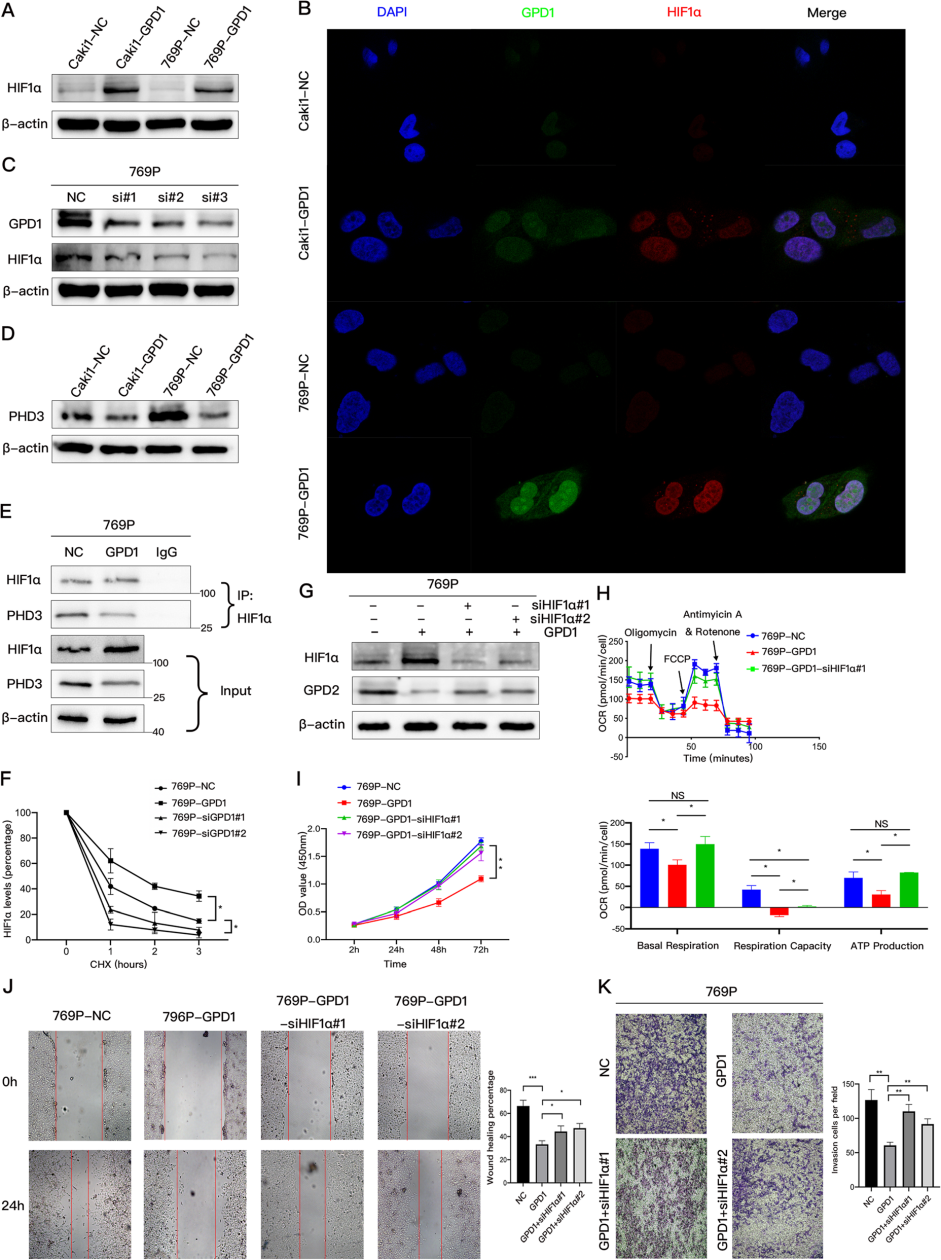
GPD1 and HIF1α form a positive feedback loop to inhibit the expression of GPD2 and mitochondrial function. (**A**) Western blotting of HIF1α in Caki1-NC, Caki1-GPD1, 769P-NC, and 769P-GPD1 cells. (**B**) Immunofluorescence stainings of GPD1 (green signal) and HIF1α (red signal) were analyzed by confocal laser scanning microscopy (magnification × 100). The nuclei of cells were stained by DAPI (blue signal). (**C**) Western blotting of GPD1 and HIF1α in 769P cells transfected with three different GPD1-specific siRNAs. (**D**) Western blotting of PHD3 in Caki1-NC, Caki1-GPD1, 769P -NC, and 769P-GPD1 cells. (**E**) Western blotting of HIF1α IP and PHD3 Co-IP in 769P-NC and 769P-GPD1 cells. (**F**) Half-lives of HIF1α in 769P-NC and 769P-GPD1 cells transfected with two different GPD1-specific siRNAs (siGPD1). Cycloheximide was used to inhibit new protein synthesis. (**G**) Western blotting of GPD2 and HIF1α in 769P-NC and 769P-GPD1 cells transfected with two different HIF1α-specific siRNAs (siHIF1α). (**H**) Oxygen consumption rate (OCR) of 769P-NC and 769P-GPD1 cells transfected with HIF1α-specific siRNAs. Baseline respiration, spare respiratory capacity, and ATP production in 769P-NC and 769P-GPD1 cells transfected with HIF1α-specific siRNAs (siHIF1α) are shown. (**I**) Cell proliferation in 769P-NC and 769P-GPD1 cells transfected with HIF1α-specific siRNAs (siHIF1α) respectively examined by CCK-8 assays. **(J-K**) Cell migration and invasion in 769P-NC and 769P-GPD1 cells transfected with HIF1α-specific siRNAs (siHIF1α) respectively examined by wound healing and Transwell assays. Data are shown as the mean + SD (*n* = 3). Statistical analysis was performed using an unpaired Student’s *t* test with a two-tailed distribution (NS *p* > 0.05, * *p* < 0.05, **p < 0.01). Abbreviations: HIF, hypoxia inducible factor; GPD1, glycerol-3-phosphate dehydrogenase 1; GPD2, glycerol-3-phosphate dehydrogenase 2; Co-IP, coimmunoprecipitation; PHD3, prolyl hydroxylase 3

HIF1α was shown to negatively regulate the expression of GPD2, so we hypothesized that GPD1 modulated HIF1α stability to inhibit the expression of GPD2. To evaluate this hypothesis, we overexpressed GPD1 in Caki1 and 769P cells, which resulted in a significant increase in HIF1α protein (Fig. 5A). Immunofluorescence indicated that the expression of HIF1α was enhanced by the overexpression of GPD1, while HIF1α was expressed both in the nucleus and cytoplasm (Fig. 5B). Further, we knocked down the expression of GPD1 using three different kinds of siRNA, which resulted in a decrease of HIF1α in 769P cells (Fig. 5C). However, overexpression of GPD1 was unable to affect the mRNA expression of *HIF1α* (Fig. S5A), indicating that GPD1 regulated the expression of HIF1α at the protein level, but not the mRNA level.

We previously performed RNA-seq on 786O-NC and 786O-GPD1 cells [14], and the results suggested that overexpression of GPD1 induces the downregulation of *PHD3* at the mRNA level. We further confirmed this downregulation at the protein level by western blotting (Fig. 5D). PHD3 is one of the three HIF prolyl hydroxylases, which includes PHD1–3. We then inferred that GPD1 reduces the degradation of HIF1α via inhibiting the expression of PHD3, which also explained why GPD1 overexpression did not change the mRNA level of *HIF1α*. Furthermore, a Co-IP assay was performed with anti-HIF1α and anti-PHD3 antibodies to confirm that overexpression of GPD1 decreased the interaction of HIF1α with PHD3 in HEK293 cells (Fig. 5E). To investigate whether GPD1 can prolong the half-life of endogenous HIF1α, we overexpressed or knocked down GPD1 in 769P cells and treated cells with a protein synthesis inhibitor, cycloheximide, for different time periods. As shown in Figs. 5F and S5B, GPD1 can significantly affect the decay of HIF1α protein and prolong the half-life of HIF1α.

Additionally, we knocked down the expression of HIF1α with two different kinds of siRNA in 769P-GPD1 cells, which led to the restoration of GPD2 expression (Fig. 5G). To further confirm this observation, we performed a seahorse assay using 769P-GPD1 cells transfected with siHIF1α, and the results showed that mitochondrial function in these cells was restored (Fig. 5H). Then CCK-8, wound healing and Transwell assays were performed, which indicated that knockdown of HIF1α significantly restored the proliferation, migration, and invasion abilities of 769P-GPD1 cells (Fig. 5I, J, and K).

### Overexpression of GPD1 inhibited lipid metabolism in ccRCC cells

**Fig. 6:**
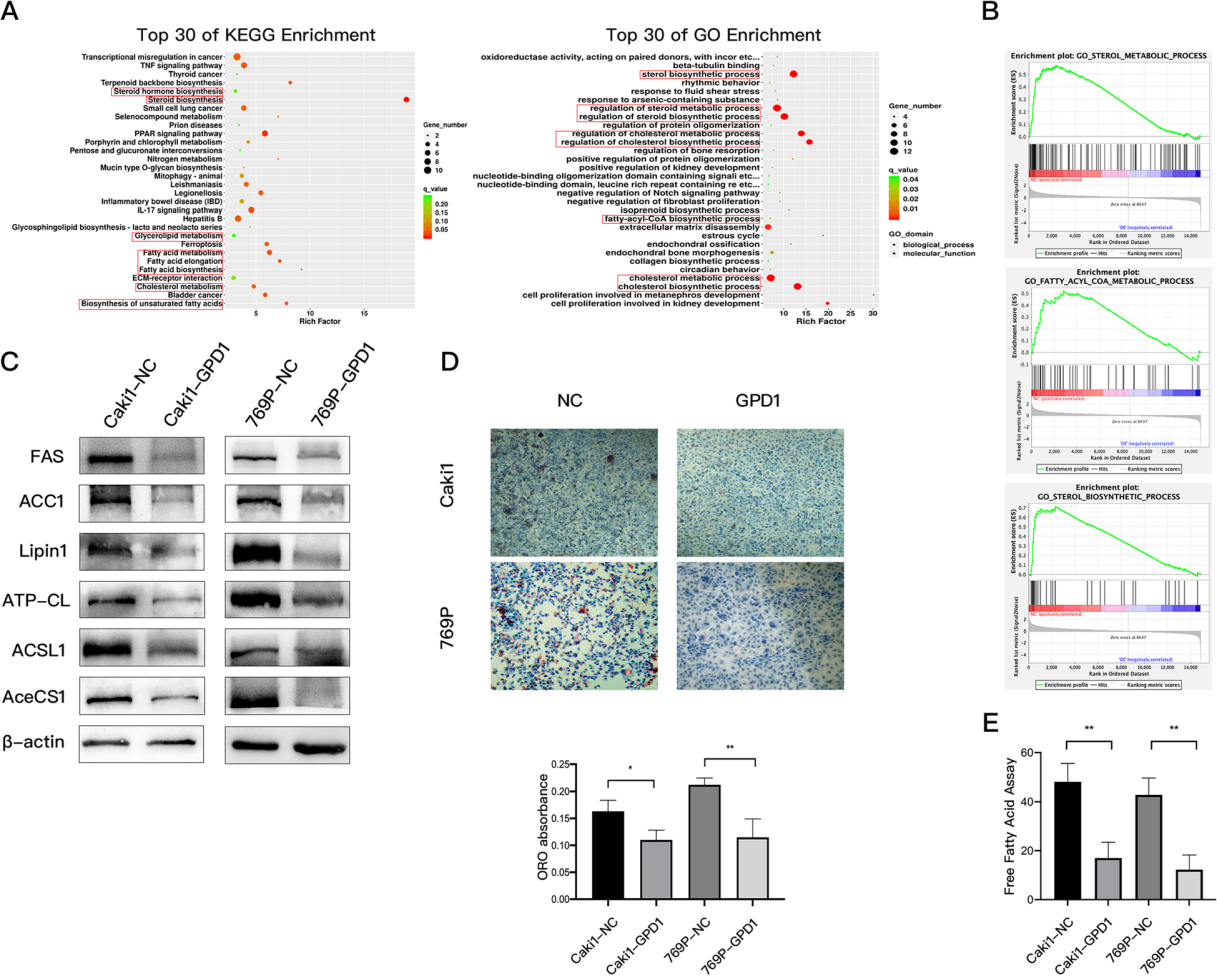
Overexpression of GPD1 inhibited lipid metabolism in ccRCC cells. (**A**) KEGG analysis and gene ontology (GO) analysis of the differentially expressed genes (|log2FC| > 1, FDR-adjusted p < 0.05) in 786O-NC and 786O-GPD1 cells. (**B**) GSEA revealed that sterol biosynthetic gene sets, fatty acyl-CoA metabolism gene sets, and sterol metabolism gene sets were less enriched in 786O-GPD1 cells (FDR < 25% and p < 0.05). (**C**) Western blotting of indicated proteins in Caki1-NC, Caki1-GPD1, 769P-NC and 769P-GPD1 cells. (**D**) Images and quantifications (at 560 nm wavelength) of Oil Red O staining in Caki1-NC, Caki1-GPD1, 769P-NC, and 769P-GPD1 cells. (**E**) Quantitation of free fatty acids in Caki1-NC, Caki1-GPD1, 769P-NC, and 769P-GPD1 cells. Data are shown as the mean + SD (n = 3). Statistical analysis was performed using an unpaired Student’s *t* test with a two-tailed distribution (* p < 0.05, **p < 0.01). Abbreviations: GPD1, glycerol-3-phosphate dehydrogenase 1; GSEA, Gene Set Enrichment Analysis; KEGG, Kyoto encyclopedia of genes and genomes; ccRCC, clear cell renal cell carcinoma; CCK-8, cell counting kit

Based on the RNA-seq data of the 786O-NC and 786O-GPD1 cells [14], KEGG and GO functional enrichment analyses were conducted to uncover the gene function and biological pathways of DEGs. From these analyses, we found that GPD1 mainly exerted its function via lipid metabolism pathways, such as fatty acid metabolism and cholesterol metabolism (Fig. 6A). Furthermore, Gene Set Enrichment Analysis (GSEA) was performed on these RNA-seq data, and the results suggested that the gene sets that positively correlated with sterol biosynthesis, fatty acyl-CoA metabolism, and sterol metabolism were less enriched in the group overexpressing GPD1 (FDR < 25% and *p* < 0.05; Fig. 6B). In addition, the gene sets that positively correlated with glycerophospholipid metabolism were more enriched in the group overexpressing GPD1 (Fig. S6A). As shown in Fig. 6C, several key proteins for fatty acid and lipid metabolism including acetyl-CoA synthetase (AceCS1), acyl-CoA synthetase (ACSL1), ATP-citrate lyase (ATP-CL), Lipin1, acetyl-CoA carboxylase (ACC1), and fatty acid synthase (FAS), were all downregulated in Caki1 and 769P cells with GPD1 overexpression. These results indicated that overexpression of GPD1 inhibited lipid metabolism, and to confirm this, a free fatty acid assay and Oil Red O staining were performed to assess the levels of free fatty acids and the accumulation of adipocyte lipid droplets, respectively. We found that the two cell lines overexpressing GPD1 showed lower levels of free fatty acids and developed lipid droplets, displaying a dramatic reduction as compared to the negative control cells under the same conditions (Fig. 6D and E). However, overexpression of GPD1 significantly increased total phospholipid levels according to a phospholipid assay (Fig. S6B).

## Discussion

ccRCC is considered to be a metabolic disease characterized by the accumulation of lipids and glycogen, which contributes to a wide variety of metabolic defects and perturbations that occur as a result of unique genetic characteristics [27]. Thus far, there have been many studies on hypoxic pathways and metabolic pathways in ccRCC. One study demonstrated that HIF2α induces hypoxia-dependent cell proliferation by activating C-MYC activity [28]. Qiu et al. reported that HIF2α-dependent PLIN2 expression promotes lipid storage, in addition to the proliferation and progression of xenograft tumors [29]. Another study found that carnitine palmitoyltransferase 1A (CPT1A), which is negatively and directly regulated by HIF1α and HIF2α at the transcriptional level, reduces the transportation of fatty acids into the mitochondria and forces the formation of lipid droplets from fatty acids for storage [2]. In this study, we discovered that HIF1α can positively regulate *GPD1* at the transcriptional level to suppress tumor progression via inhibiting mitochondrial function and lipid metabolism. Our data revealed a new mechanism by which the hypoxic and lipid metabolism pathways are involved in the progression of ccRCC.

**Fig. 7:**
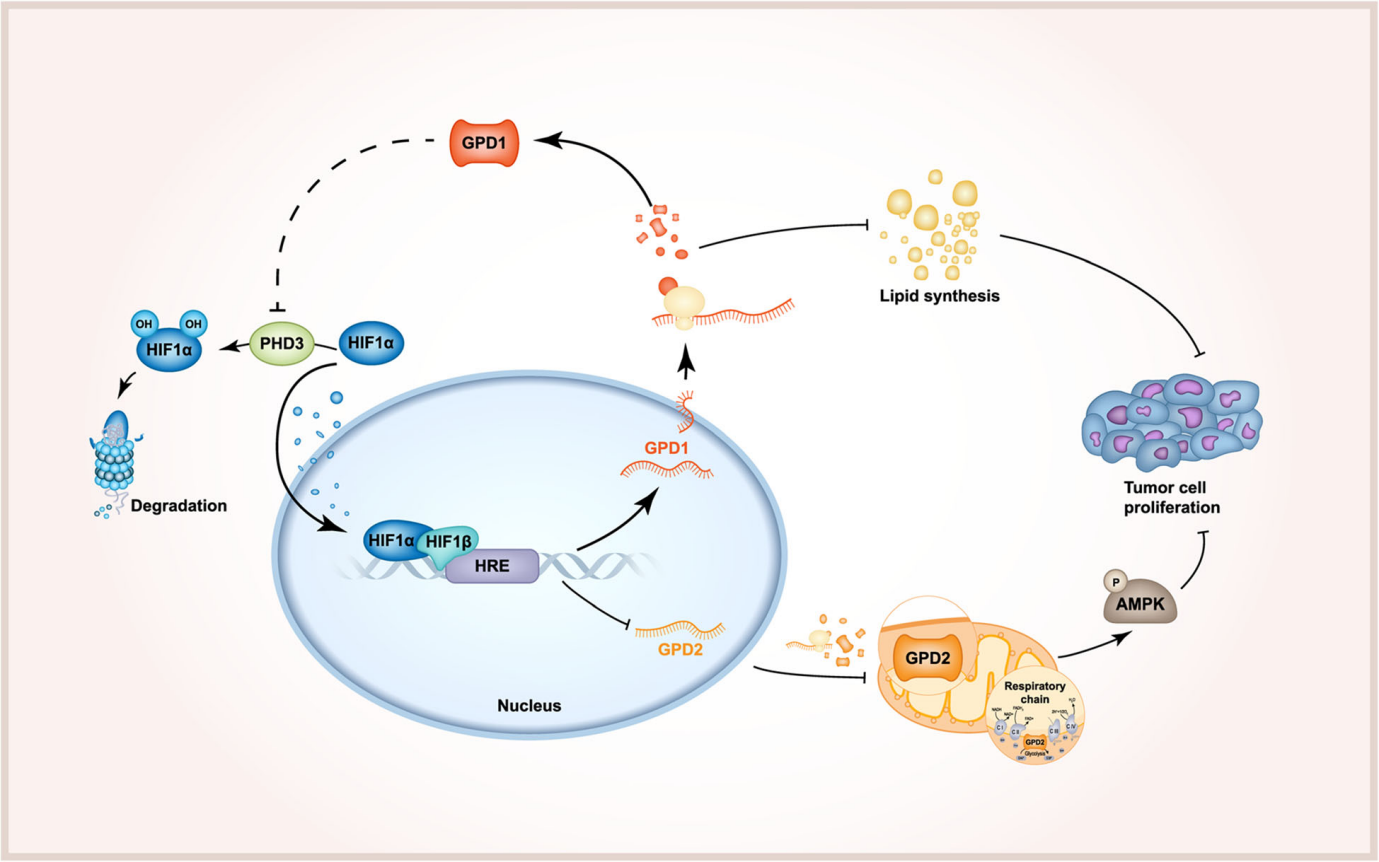
Schematic diagram of the HIF1α-GPD1 feedforward loop

Madiraju et al. found that metformin can inhibit gluconeogenesis by inhibiting GPD2 to induce AMPK phosphorylation and proposed that GPD2 may be a target for tumor therapy [12, 30]. Subsequent studies support this view in tumors, including thyroid cancer [13], prostate cancer [31], and lung cancer [14]. We are the first to propose that in ccRCC, GPD1 can suppress the expression of GPD2, thereby inhibiting mitochondrial function and inducing AMPK phosphorylation. We confirmed that HIF1α can negatively regulate the expression of GPD2 at the transcriptional level, thereby inhibiting tumor progression, which is new mechanism-of-action for HIF1α as a tumor suppressor. In addition, GPD1 can also stabilize the expression of HIF1α by inhibiting the expression of PHD3 and reducing the degradation of HIF1α. Therefore, GPD1 can form a positive feedback loop with HIF1α to regulate GPD2 expression and mitochondrial function (Fig. 7), but the specific mechanism by which GPD1 affects PHD3 needs further study.

Bioinformatic analysis showed that GPD1 may play an essential role in the lipid metabolism pathway. Another study found that GPD1 mutations can lead to hypertriglyceridemia and a fatty liver in infants [32]. Our RNA-seq analysis and experiments also confirmed that overexpression of GPD1 led to the significant inhibition of lipid metabolism pathways. However, glycerol-3-phosphate (G3P), a product catalyzed by GPD1, should increase after the overexpression of GPD1, which provides more substrates for the synthesis of triglyceride. We speculate that increased G3P is more inclined to produce glycerophospholipids, which leads to a decrease in the production of lipids including triglycerides and fatty acids. Some research has shown that ccRCC patients have lower levels of phospholipids, and that the phosphatidylethanolamine (PE) synthesis pathway is suppressed in cancerous tissues, which is associated with cell proliferation [3, 8]. The results of GSEA and phospholipid assays (Figs. S6A and S6B) support this speculation, but the mechanism needs more research. Our previous studies have found that in prostate cancer and lung cancer, GPD1 can enhance the anti-tumor effect of metformin, with G3P playing a major role in inhibiting tumor proliferation [14]. Thus, we believe that G3P may have an instantaneous effect, because exogenous G3P was increased in a short period of time, while GPD2 inhibition was a long-term effect, as the activation of the hypoxic pathway required a longer process.

Clinically, detecting GPD1 expression can be used for the early diagnosis of ccRCC and provide guidance for the prognosis of the disease. In addition, based on the role of GPD1 in tumor proliferation and migration, it is possible to find a drug that binds the enzyme receptor at its binding site and promotes the expression of GPD1, which could provide a novel therapy for ccRCC, but this requires further study.

## Conclusions

In summary, our study confirmed that GPD1 forms a positive feedback loop with HIF1α to decrease the expression of GPD2, a key target involved in mitochondrial oxidative phosphorylation. This results in the inhibition of mitochondrial function, induction of AMPK phosphorylation, and suppression of lipid metabolism (Fig. 7). This study revealed a new mechanism underlying the tumor suppression ability of HIF1α and provides insights into the role of hypoxia and lipid metabolism in tumor progression. The results of this study have the potential to be used in the development of novel therapies for ccRCC.

## Supplementary Information

**Additional file 1.**

## Data Availability

The datasets used and/or analyzed during this study are available from the corresponding author on reasonable request.

## Abbreviations

ccRCC: clear cell renal cell carcinoma
GPD1: Glycerol-3-phosphate dehydrogenase 1
HIF: Hypoxia inducible factor
IHC: Immunohistochemical
TMA: Tissue microarray
qRT-PCR: quantitative real-time PCR analysis
TCGA: The Cancer Genome Atlas
CCK-8: Cell counting kit
