# Supplementary material for: A HIF1α-GPD1 feedforward loop inhibits the progression of renal clear cell carcinoma via mitochondrial function and lipid metabolism
Source: J Exp Clin Cancer Res. 2021 Jun 7;40:188. doi: 10.1186/s13046-021-01996-6 (PMC8185942; doi:10.1186/s13046-021-01996-6)

**Supplementary Materials**

**Reagents and assay kit**

RPMI 1640 medium, DMEM medium, Fetal Bovine Serum (FBS), Penicillin and streptomycin and Fetal Bovine Serum were purchased from Gibco (Gibco, USA). F-12K medium, McCoy's 5a medium, Eagle's Minimum Essential medium and Leibovitz's L-15 medium were purchased from American Type Culture Collection (Manassas, VA, USA). Glutaraldehyde was obtained from Guangzhou Kinged Medical Diagnostics center. 1, 1-Dimethylbiguanide hydrochloride was purchased from Sigma Aldrich (D150959-5G, Sigma Aldrich, MO, USA). CCK-8 counting kit was purchased from Beyotime Biotechnology (C0038, Beyotime Biotechnology, shanghai, China). XFe24 Cell Mito Stress Test Kit and XF ATP Rate Assay was purchased from Agilent Technologies.

**The sequences of all the primers**

| **Name** | **Oligonucleotide sequence (5' - 3')** | |
| --- | --- | --- |
|  | **Forward** | **Reverse** |
| **SMTNL2** | CCCCTGAGATTGCCCAAAACT | CATGGGTGATAGAGCCGCAG |
| **ANK** | GAAGATGCAATGACCGGGGA | CTAAAGCCCATGTAACCCTCTG |
| **WDR72** | TGATGACCAGCGAACGATTGT | CACGCACTGTCCATTGGTG |
| **LHFPL3** | CCTACTGGATAGGCGACGG | GAGGCCGATAAAGAAGGAGGC |
| **EMX2** | CGGCACTCAGCTACGCTAAC | CAAGTCCGGGTTGGAGTAGAC |
| **HMGCS2** | GACTCCAGTGAAGCGCATTCT | CTGGGAAGTAGACCTCCAGG |
| **TRNP1** | CGGCTGGAAGGACTACGGAT | GAGTTGGAAGGAGCTCAGCC |
| **CCDC64** | GAGCTGGAGAGTGATGTGAAGC | TTGGTTCTGTTCCGACAGTTC |
| **TMEM125** | CTGCTGTATCAAGTGGGTGTG | GATGTGGTCTCGTGACGCC |
| **SLC6A19** | TACCTGGAGTTCGCCATCG | GTCCCACCATGAAGGACGTG |
| **SMIM24** | TTCCAAGGCCAGGGCTGA | AGGCAGCCAGGAATCTTCAC |
| **FLRT3** | CCTCATCGGGACTAAAATTGGG | ATGGATGTCAGAAAGCGATCATT |
| **GPD1** | GCCATCTGAAGGCAAACGC | GCCAATGGTTGTCTCACAGAAC |
| **UGT3A1** | CAAGGAGCATTTCCCAGAAGG | TCTCAGTGGTTCAGGATACACTC |
| **ABP1** | CCTAAGCAACCAAGAGCTGAA | CGGTGACATTGGGATGCTCC |
| **GPD2** | GGCAGTGAAAGGGACGATTCT | GCTGCTTTAACATAGGCCAGGT |
| **PHD3** | CTGGGCAAATACTACGTCAAGG | GACCATCACCGTTGGGGTT |
| **Β-actin** | AGCGAGCATCCCCCAAAGTT | GGGCACGAAGGCTCATCATT |
| **HIF1α** | GAACGTCGAAAGAAAAGTCCTG | CCTTATCAAGATGCGAACTCACA |
|  |  |  |

**The antibodies of all proteins**

| Name | Description | Product code | Company |
| --- | --- | --- | --- |
| anti-GPD1 | mouse monoclonal | Sc-376219 | Santa Cruz Biotechnology, USA |
| anti-GPD2 | rabbit monoclonal | Ab182144 | Abcam, USA |
| anti-AMPK | rabbit monoclonal | #2532 | CST |
| phospho-AMPK | rabbit monoclonal | #2535 | CST |
| anti-S6K1 | rabbit monoclonal | Ab32529 | Abcam, USA |
| Phospho-p70 S6 Kinase | rabbit monoclonal | #9234 | CST |
| PCNA | rabbit monoclonal | #13110 | CST |
| Anti-MMP2 | rabbit monoclonal | #40994 | CST |
| Bcl-2 | mouse monoclonal | #15071 | CST |
| Anti-mTOR | rabbit monoclonal | Ab134903 | Abcam, USA |
| Phospho-mTOR | rabbit monoclonal | Ab109268 | Abcam, USA |
| Beta-actin | mouse monoclonal | BM0627 | BOSTER, China |
| PHD3 | rabbit polyclonal | 18325-1-AP | Proteintech, China |
| HIF1α | mouse monoclonal | Ab1 | Abcam, USA |
| HIF2α | rabbit monoclonal | #59973 | CST |
| Fatty Acid and Lipid Metablolism Antibody Sampler Kit | rabbit monoclonal | #8335 | CST |

**The following sequences were used for siRNA experiments:**

| **NAME** | **sequence (5' - 3')** |
| --- | --- |
| Negative control | UUCUCCGAACGUGUCACGUTT |
| GPD1-Homo-558 | CCCAGGGACAACUCCUGAATT |
| GPD1-Homo-433 | GAGGCCGATAAAGAAGGAGGC |
| GPD1-Homo-211 | GCUCAUCUCGGAAGUGAUUTT |
| GPD2-Homo-494 | GCAUUUCAGAACCAGUUAATT |
| GPD2-Homo-958 | GCCCUUGAACAUUUCCCAATT |
| GPD2-Homo-1845 | GCAUCUUGCCGCCACCUAUTT |
| HIF1α-homo-1217 | GCCGCUCAAUUUAUGAAUATT |
| HIF1α-homo-1612 | GCUGGAGACACAAUCAUAUTT |
| HIF2α-homo-718 | CUCCUCAGUUUGCUCUGAATT |
|  |  |
|  |  |
|  |  |


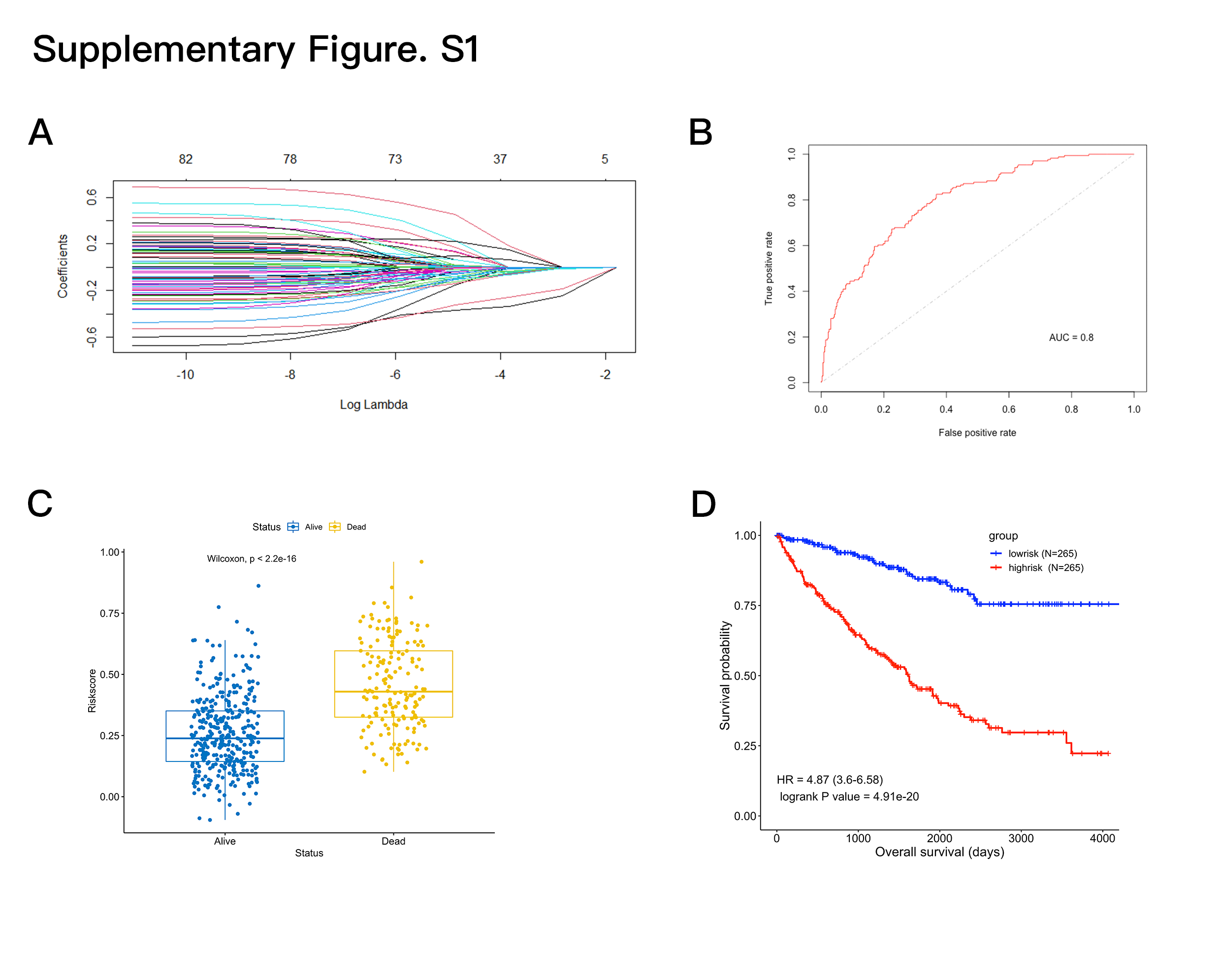


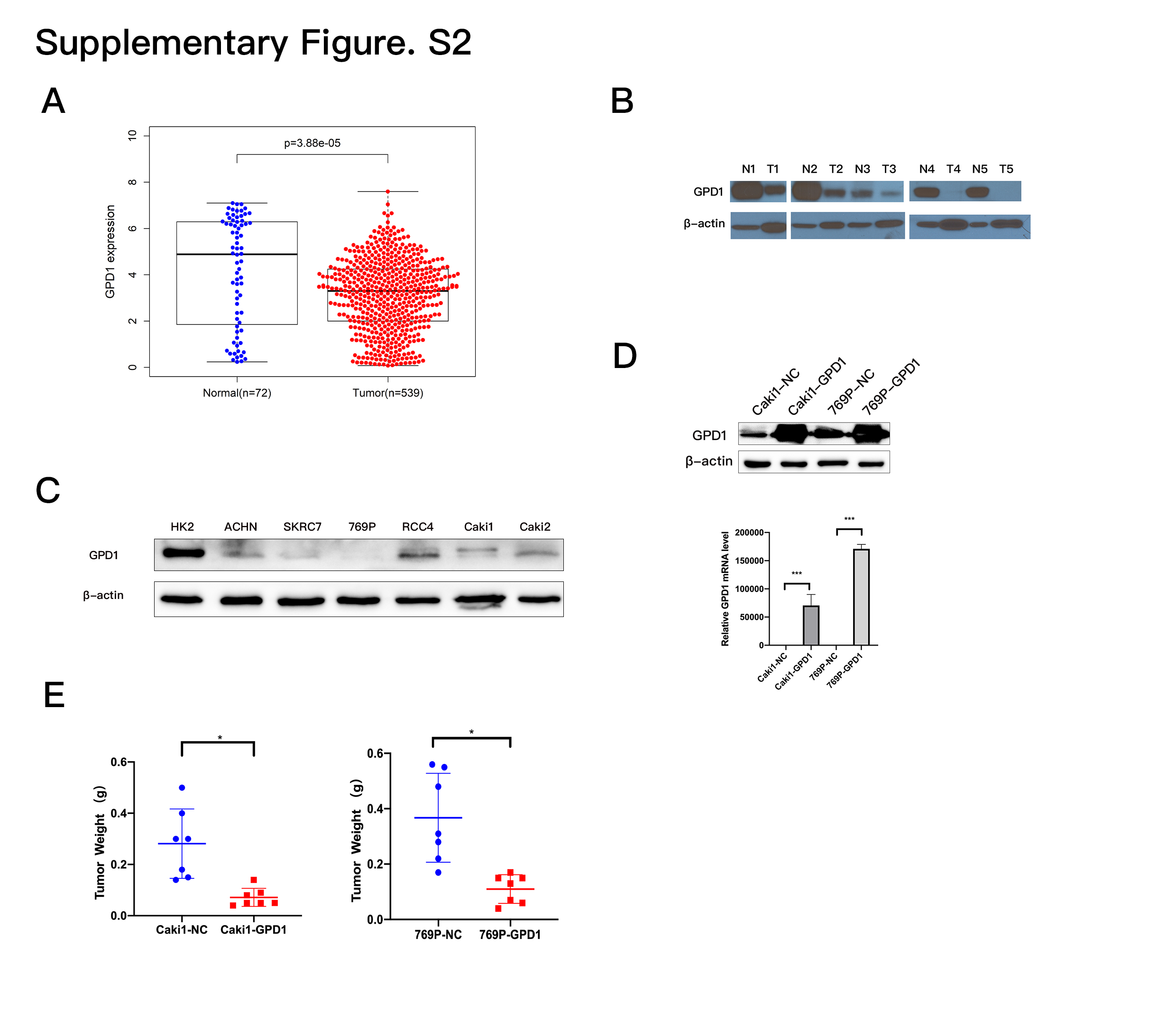


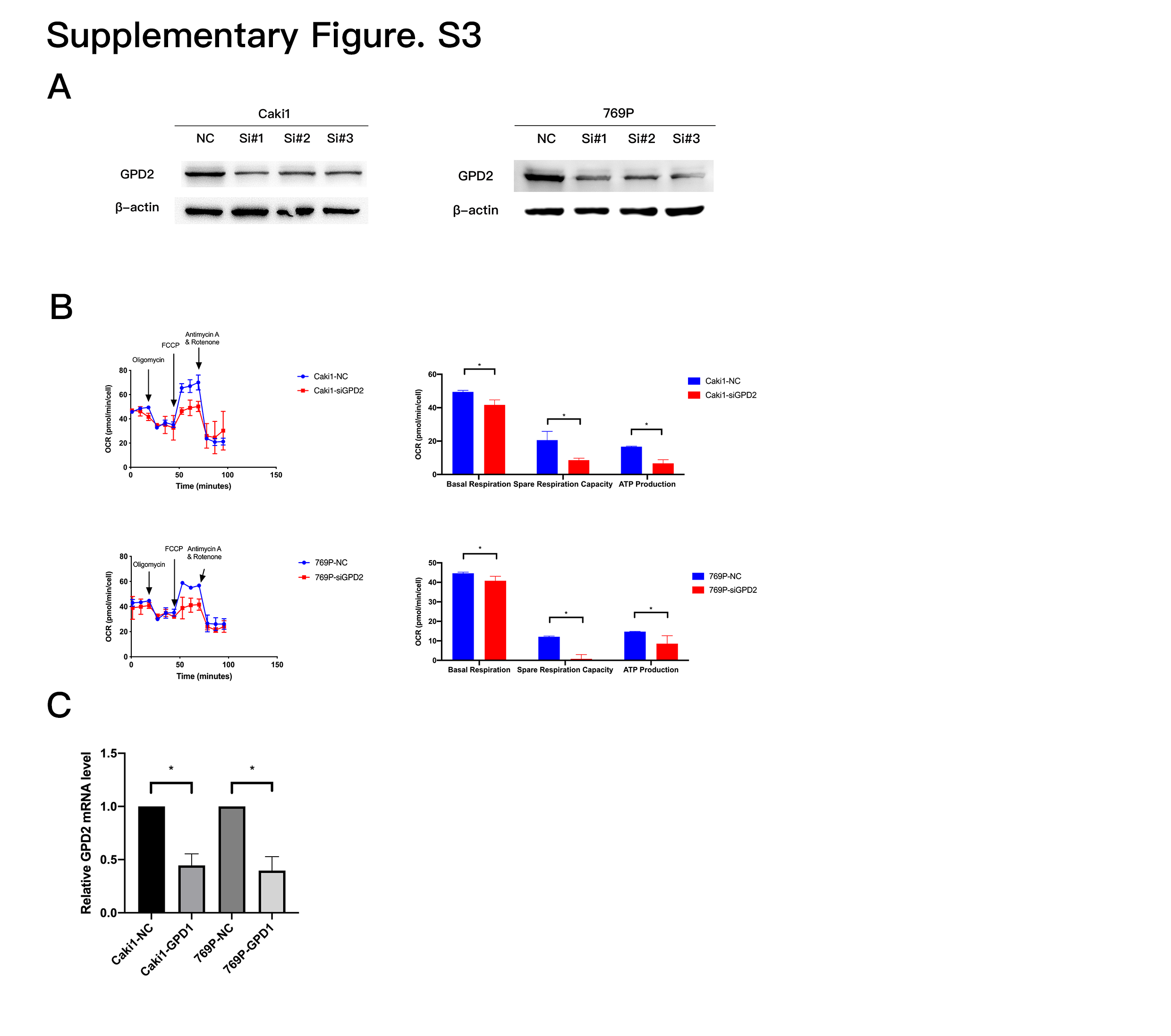

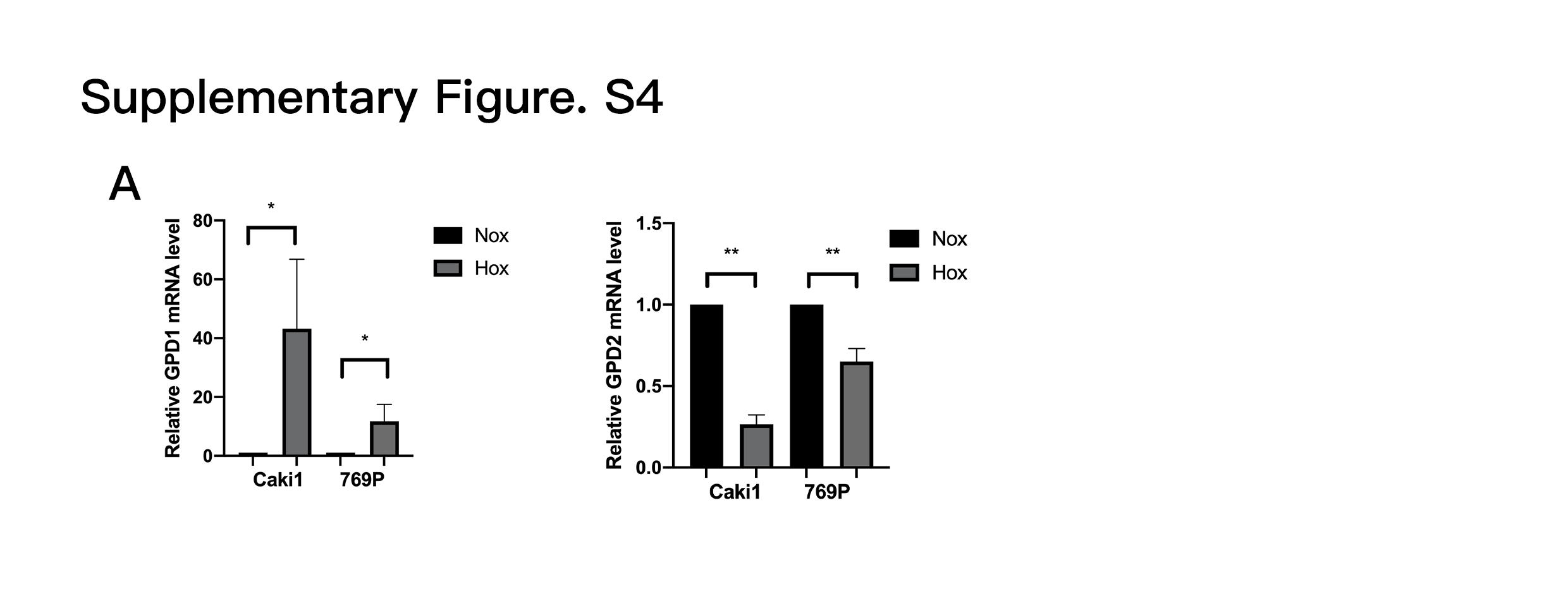

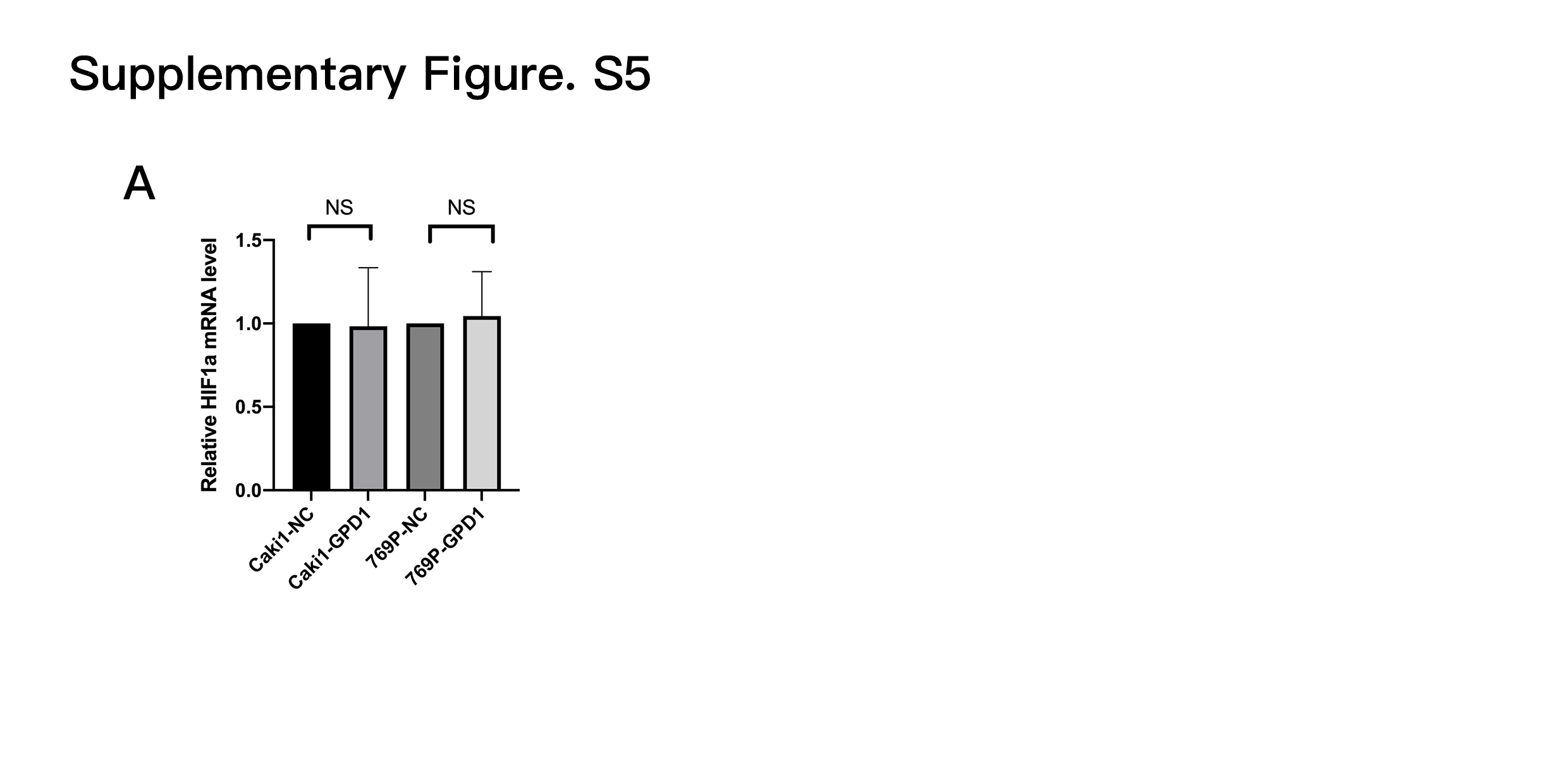

Supplement: Supplementary file 1 — Additional file 1. [file 13046_2021_1996_MOESM1_ESM.docx]
